# The preablation monocyte/ high density lipoprotein ratio predicts the late recurrence of paroxysmal atrial fibrillation after radiofrequency ablation

**DOI:** 10.1186/s12872-020-01670-3

**Published:** 2020-09-07

**Authors:** She-an Chen, Man-man Zhang, Meifang Zheng, Fei Liu, Lei Sun, Zheng-yu Bao, Fu-kun Chen, Hong-xiao Li, Xiang Gu

**Affiliations:** 1 Clinical Medical College, Yangzhou University, Yangzhou, Jiangsu China; 2 Department of Cardiology, Northern Jiangsu People’s Hospital, No. 98, Nantong West Road, Yangzhou, 225001 Jiangsu China; 3 Clinical Medical College, Dalian Medical University, Dalian, 116044 Liaoning China

**Keywords:** Paroxysmal atrial fibrillation, Radiofrequency ablation, Monocyte/HDL ratio, Left atrial diameter, Late recurrence

## Abstract

**Background:**

The monocyte/high-density lipoprotein ratio (MHR) has emerged as a promising alternative biomarker in the fields of cardiovascular disease and atrial fibrillation (AF). This retrospective study was aimed to explore the predictive value of the MHR for the late recurrence of AF after radiofrequency ablation.

**Methods:**

From April 2015 to October 2018, patients with paroxysmal AF who had undergone radiofrequency catheter ablation at Subei People’s Hospital of Jiangsu Province were enrolled in our study. All the participants were observed until November 2019 after the procedure. During the postoperative follow up, the patients were categorized into the recurrence group and maintenance of sinus rhythm group based on who had experienced AF recurrence.

**Results:**

One hundred twenty-five patients were diagnosed with paroxysmal AF, with an average age of 61.2 ± 9.3 years. Forty-seven patients had developed late recurrence during a mean follow up of 25.1 ± 12.0 months. The AF recurrence event rates were significantly increased in the highest MHR tertile compared with those in the lowest MHR tertile (22.0% vs. 57.1%; *P* < 0.05). On multivariate logistic regression analysis, the preablation MHR (*OR* = 1.34; 95% *CI* = 1.12 ~ 1.60; *P* = 0.001) and left atrial diameter (LAD) (*OR* = 1.21, 95% *CI* = 1.08 ~ 1.35; *P* = 0.001) were independent risk factors predicting the recurrence of AF after radiofrequency ablation. Furthermore, receiver operating characteristic (ROC) curve analysis revealed that the area under the curve (AUC) of the MHR was 0.712 (95% *CI* = 0.618 ~ 0.806; *P* = 0.000) and that of LAD was 0.739 (95% *CI* = 0.653 ~ 0.814; *P* = 0.000). Z-test found no significant difference between the MHR and LAD regarding the AUC (*Z* = 0.451; *P* = 0.652).

**Conclusion:**

An elevated preablation MHR was associated with an increased risk of the postoperative recurrence of AF. Additionally, the MHR independently predicted the late recurrence of paroxysmal AF after radiofrequency ablation, with the same predictive value as LAD.

## Background

Atrial fibrillation (AF) is a common tachyarrhythmia, accompanied by disorder of atrial electrical activity and mechanical dysfunction. On account of the gradual aging of the population and unhealthy living habits, the global incidence of AF is expected to escalate over the next few decades. During the attack of AF, part of the blood is stagnated in the atrium, and the slow blood flow easily contributes to thrombus formation in the atrium. Therefore, patients with AF are more likely to have a high incidence of stroke and systemic embolism, seriously affecting the quality of life of patients and resulting in a huge burden to public health [1].

Pulmonary vein electrical isolation achieved by radiofrequency catheter ablation has been the cornerstone of catheter-based therapies for AF, with the greatest efficacy as a promising treatment option in patients with paroxysmal AF [2]. The techniques of catheter ablation for AF have undergone a profound evolution recently. Nonetheless, long-term postoperative atrial arrhythmia-free survival remains unsatisfactory, with a high AF recurrence rate of 25–50% during the postoperative follow up [3]. Therefore, it is of great significance to detect the clinical factors influencing the successful maintenance of the sinus rhythm in patients with AF after ablation.

The monocyte/HDL ratio (MHR), as a new indicator of inflammation and oxidative stress, has been widely explored in the field of cardiovascular disorders, including acute coronary syndrome [4]. Additionally, the MHR was found to be a candidate cardiovascular prognostic marker in chronic kidney disease [5]. Furthermore, the MHR could be used as a predictor of AF recurrence after cryoballoon-based catheter ablation and an elevated preablation MHR was associated with increased recurrence of AF [6]. However, the prognostic value of the MHR in patients with paroxysmal AF after radiofrequency ablation remains controversial. Thus, the study was aimed to investigate the prognostic impact of the MHR on paroxysmal AF after radiofrequency ablation in an observational cohort.

## Methods

### Study population

In this retrospective study, patients with paroxysmal AF who were admitted for pulmonary vein isolation (PVI) treatment using radiofrequency ablation at the Department of Cardiology of Subei People’s Hospital of Jiangsu Province from April 2015 to October 2018 were enrolled. All the participants had failed antiarrhythmic drugs previously, and no thrombus was found in the left atrium before surgery. Additionally, patients who had met the following criteria were excluded from the present study: rheumatic heart valve disease, left atrial thrombus, LAD> 55 mm, uncontrolled thyroid dysfunction, congenital heart disease, liver and kidney dysfunction, blood system diseases, contraindication to anticoagulation, and malignant tumors with a life expectancy less than 1 year. The preprocedural informed consent was obtained from all the involved participants, and the study was conducted according to the Helsinki Declaration approved by the ethics committee of Northern Jiangsu People’s Hospital.

### Catheter ablation procedure and postoperative follow up

The ablation procedure details had been fully described in the study published in *Europace* [7]. In our study, under the guidance of the three-dimensional mapping system, Ensite3000 Navx (St. Jude Medical) or CARTO system (Biosense Webster, Inc), electrical isolation of the circumferential pulmonary vein lesions was performed by well-experienced professors. Additional ablation lines of the left atrium roof should also be implemented in some patients as necessary. The endpoint of the ablation was the absence or dissociation of potentials between the pulmonary vein and left atrium. Additionally, the electrical block of the pulmonary veins was repeatedly evaluated 30 min after the initial isolation. All the patients took direct oral anticoagulants with warfarin at least 4 weeks before and 3 months after surgery, with a target international normalized ratio of 2.0–3.0. Additionally, all the patients took amiodarone hydrochloride or class IC antiarrhythmic drugs for more than 1 month after ablation. The postoperative follow-up schedules were performed at 3, 6, and 12 months after the surgery and every 6 months thereafter, or whenever they developed discomfort symptoms consistent with recurrent AF. A 48-h Holter electrocardiogram or 12-lead electrocardiogram was recorded at the outpatient follow-up visits. The blanking period was defined as the first 3 months after the AF ablation procedure. Beyond the blank period, AF recurrence referred to the detection of AF/atrial tachycardia attack with a duration >30 s, as assessed by electrocardiographic monitoring.

### Data collection

The clinical data of all the patients with AF before ablation were collected and included basic demographic characteristics, disease history, AF duration, the CHA2DS2-VASc score, the HATCH score, the monocyte count and other items within the complete blood count, creatinine and uric acid, Cystatin C, D-Dimer, lipid protein profile, left atrial diameter (LAD), left ventricular ejection fraction (LVEF), and follow-up time. The monocyte/HDL ratio (MHR) was calculated as the monocyte count divided by the HDL. All the blood samples were collected 24 h before the procedure.

### Statistical analysis

All the data were analyzed using SPSS software, version 20.0 for Windows (SPSS Inc., Chicago, Illinois, USA). The Kolmogorov–Smirnov criterion was used to assess normality. Continuous variables with a normal distribution were presented as the means ± standard deviation; otherwise, they were described as medians (interquartile range). The categorical variables were summarized as frequencies and percentages. Comparisons of two continuous variables with a normal distribution were carried out by independent samples Student’s t-test, whereas comparisons between two continuous variables were implemented using the Mann–Whitney U test. Categorical variables were compared using chi-squared test or Fisher’s exact test. Univariate and multivariate binary logistic regression analyses using the backward likelihood ratio method were employed to determine the risky predictors of AF recurrence. The correlations were assessed using Spearman’s rank test. Receiver operating characteristic (ROC) curve analysis was used to assess the predictive value of the risk factors to predict AF recurrence. A two-tailed *P* value < 0.05 was considered statistically significant.

## Results

### Baseline characteristics and demographical features

**Fig. 1:**
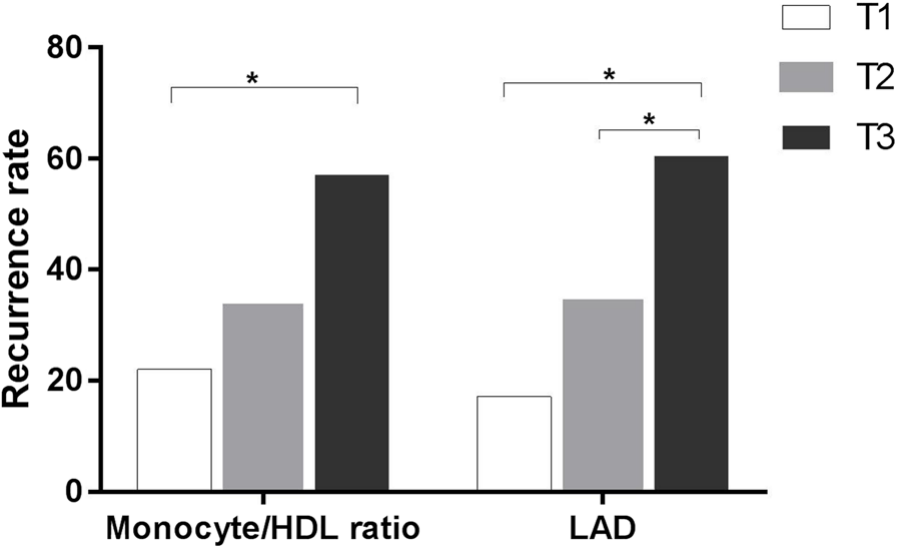
Percentage of the patients developing post-ablation AF recurrence stratified by tertiles of pre-ablation Monocyte/HDL ratio and LAD

One hundred twenty-five patients with paroxysmal AF were enrolled, including 69 males and 56 females, with an average age of 61.2 ± 9.3 years. Forty-seven patients had developed late recurrence of AF during a mean follow up of 25.1 ± 12.0 months. Depending on the postoperative recurrence of AF, the patients were divided into the recurrence group and maintenance of sinus rhythm group. The baseline demographic characteristics and demographical features, laboratory, and procedural details of both cohorts are summarized in Table 1. The two groups were significantly different in terms of the AF duration, history of diabetes mellitus, body mass index, D-dimer level, monocyte count, HDL cholesterol level, monocyte/HDL ratio, and LAD (*P* < 0.05). Furthermore, the patients were stratified into 3 groups according to the MHR tertile (T1: < 5.68; T2: 5.68–8.29; T3:≥8.29) and subgroup analysis showed that patients in T3 had a higher rate of AF recurrence than those in T1 (22% vs. 57.1%; *P* < 0.05; Fig. 1). Additionally, subgroup analyses stratified by LAD tertile (T1: < 33.96 mm; T2: 33.96–38 mm; T3: > 38 mm) revealed that the rate of recurrence was significantly higher in the highest T3 group than that in the T1 and T2 groups (*P* < 0.05; Fig. 1).

**Table 1:** Baseline characteristics and demographical features of the study population

Variables	sinus rhythm (*n* = 78)	AF recurrence (*n* = 47)	*P* value
Age (years)	61.5 (13.25)	63.0 (15.0)	0.717
Gender (female)	38 (48.7%)	18 (38.3%)	0.256
Body mass index (kg/m^2^)	24.7 ± 2.9	26.1 ± 3.1	0.016*
Hypertension	47 (60.3%)	34 (72.3%)	0.171
Diabetes mellitus	9 (11.5%)	14 (29.8%)	0.011*
Coronary artery disease	19 (24.4%)	9 (19.1%)	0.499
Stroke	13 (16.7%)	8 (17.0%)	0.959
AF duration (months)	12.0 (31.25)	24.0 (48.0)	0.035*
Procedure time (minnutes)	240 (60.0)	240 (119.0)	0.969
CHA2DS2-VASc score	2.0 (2.0)	2.0 (2.0)	0.172
HATCH score	0.95 ± 0.89	1.09 ± 0.93	0.418
Hemoglobin (g/L)	137.9 ± 21.6	137.1 ± 16.2	0.847
AST (U/L)	21 (10.25)	23 (8.0)	0.578
Serum creatinine (umol/L)	83.0 ± 18.3	86.6 ± 19.1	0.301
Uric acid (umol/L)	309.3 ± 84.5	325.2 ± 96.4	0.336
WBC (×10^9^/L)	5.40 (1.91)	5.69 (2.35)	0.359
Monocyte (× 10^9^/L)	0.32 (0.13)	0.35 (0.15)	0.007*
LDL cholesterol (mmol/L)	2.42 (1.18)	2.36 (0.88)	0.521
HDL cholesterol (mmol/L)	1.33 (0.54)	1.09 (0.40)	0.002*
Monocyte/HDL ratio	6.34 (3.39)	8.29 (4.99)	0.000*
Cystatin C (mg/L)	0.8 (0.39)	0.8 (0.30)	0.165
D-Dimer (mg/L)	0.27 (0.21)	0.27 (0.14)	0.008*
LAD (mm)	34 (6.0)	38 (6.0)	0.000*
LVEF (%)	62 (1.25)	61 (3.0)	0.205
Follow-up duration (months)	22 (20.25)	19 (12.0)	0.165

*P < 0.05

### Predictors of AF recurrence

**Fig. 2:**
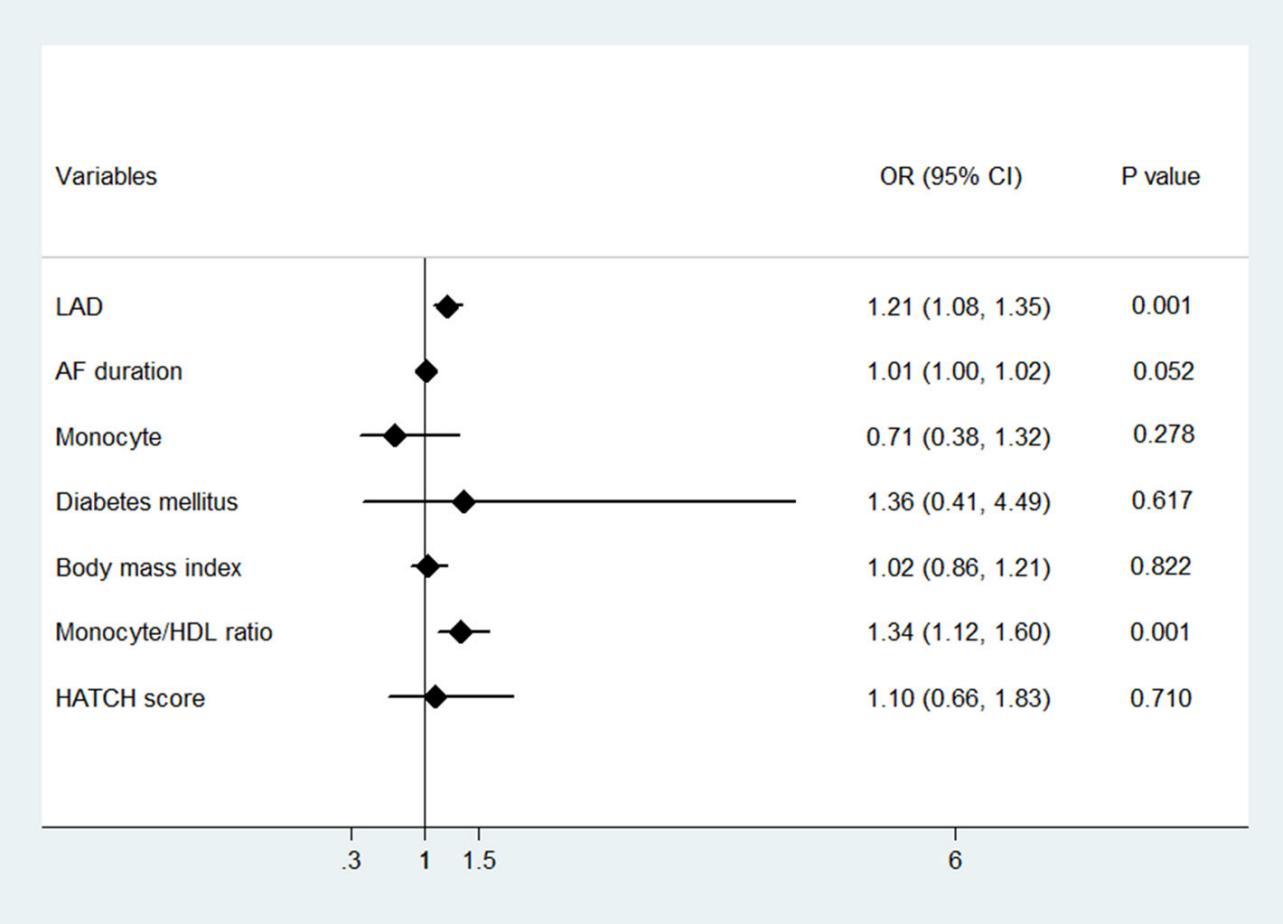
Forest plot of multivariate logistic regression modeling results of the AF recurrence after ablation

According to univariate binary logistic analysis, AF duration, history of diabetes mellitus, body mass index, monocyte count, the monocyte/HDL ratio, and the LAD were significantly associated with AF recurrence (*P* < 0.05). However, the CHA2DS2-VASc score, HATCH score, and D-dimer level were identical to those of the sinus rhythm (Table 2). Furthermore, we performed multivariate logistic regression analysis using the backward likelihood ratio method, and the analysis revealed that the LAD (*OR* = 1.21; 95% *CI* = 1.08 ~ 1.35; *P* = 0.001) and preablation MHR (*OR* = 1.34, 95% CI = 1.12 ~ 1.60, *P* = 0.001) were independent risk factors predicting the recurrence of AF after radiofrequency ablation. (Fig. 2).

**Table 2:** Univariate logistic regression modeling results of the AF recurrence

Variables	Univariate model		
	*OR*	95% *CI*	*P*
LAD	1.250	1.125–1.389	0.000*
AF duration	1.012	1.001–1.023	0.026*
Monocyte	1.691	1.167–2.451	0.005*
HDL cholesterol	0.164	0.049–0.543	0.003*
D-Dimer	4.560	1.0–20.794	0.050
Diabetes mellitus	3.253	1.278–8.281	0.013*
Body mass index	1.166	1.026–1.326	0.019*
CHA2DS2-VASc score	1.214	0.924–1.597	0.164
HATCH score	1.180	0.793–1.755	0.415
Monocyte/HDL ratio	1.377	1.176–1.614	0.000*

*P < 0.05

### Predictive value of risk factors

**Fig. 3:**
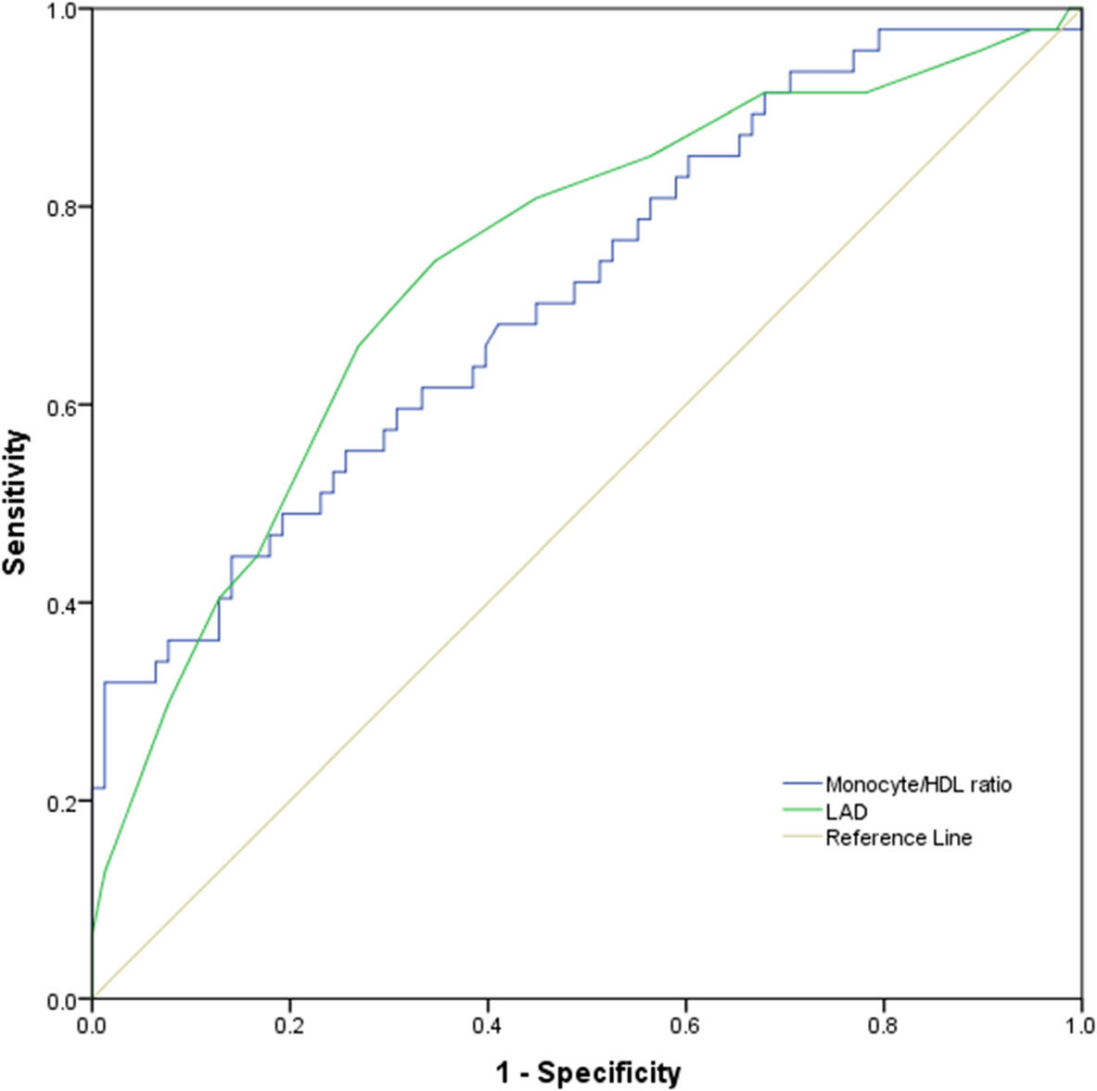
Receiver operating characteristic curve of pre-ablation variables for predicting AF recurrence after ablation

The predictive value of risk factors to predict the recurrence of AF after ablation was evaluated by ROC analysis. The areas under the curve (AUC) of the MHR and LAD were 0.712 (95% *CI* = 0.618 ~ 0.806; *P* = 0.000) and 0.739 (95% *CI* = 0.653 ~ 0.814 *P* = 0.000), respectively (Fig. 3). Furthermore, Z-test showed no significant difference between the MHR and LAD concerning AUC (*Z* = 0.451; *P* = 0.652).

**Fig. 4:**
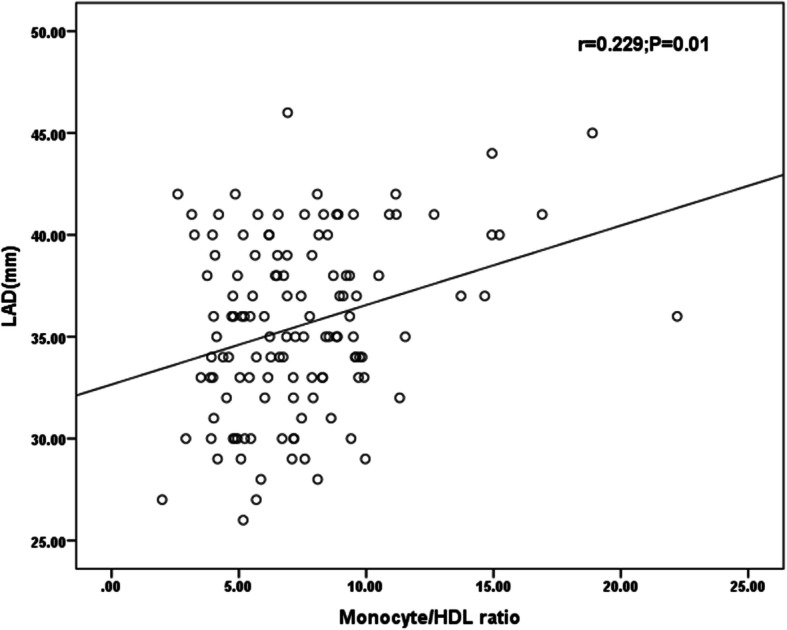
Correlation of the preablation monocyte/HDL ratio with LAD (r = 0.229, *P* = 0.01)

Additionally, correlation analysis revealed a positive correlation of the MHR with the LAD (*r* = 0.229; *P* = 0.01; Fig. 4).

## Discussion

In our retrospective study, we focused more on the relationship between the preprocedural MHR and development of AF recurrence after radiofrequency ablation. The clinical data of 125 patients of paroxysmal AF treated by circumferential pulmonary vein electrical isolation with an average follow-up time of 25.1 ± 12.0 months was systematically reviewed. We found that the preablation LAD and MHR were risk factors affecting the therapeutic effect of radiofrequency ablation. Additionally, we had demonstrated that the MHR along with LAD is an independent prognostic factor of AF recurrence in patients with paroxysmal AF who had undergone a single catheter ablation procedure.

AF is the most common atrial arrhythmia in clinical practice, which seriously affects the quality of life of patients and may cause some serious complications, such as heart failure and stroke. Presently, radiofrequency catheter ablation is an effective methods to treat AF. The success rate of the radiofrequency ablation of paroxysmal AF is between 70 and 80% in 1 year [8] However, some patients will develop relapse of AF after ablation. Many factors, including the AF duration, LAD, and left ventricular dysfunction, have been reported to be related to the recurrence of AF after radiofrequency ablation. The pathophysiological mechanism of AF has not been clearly revealed yet, and many underlying mechanisms participate in the recurrence and maintenance of AF. In recent years, emerging of evidence has revealed that the cross-linked process of inflammation and oxidative stress may result in atrial fibrosis, which is probably the main constituents of AF pathophysiology [9–11] and may play a role in substrate modification by electrical and structural remodeling of the atrium, consequently increasing the susceptibility of AF [12]. Compared with patients with sinus rhythm, the levels of the markers of inflammation and oxidative stress, such as C-reactive protein and interleukin-6, in patients with AF were significantly increased [13]. Inflammation and oxidative stress promote the occurrence of AF, while inhibition of the inflammatory response could reduce the risk of AF. A meta-analysis of 50 randomized controlled studies [14] revealed that the prophylactic use of corticosteroids to inhibit the inflammatory response significantly reduced the incidence of postoperative AF in 3323 patients who had undergone cardiac surgery.

As a typical inflammatory cell, monocytes mediate the process of inflammation and oxidative stress by binding to adhesion molecules expressed on damaged vascular endothelial cells and inducing the production of various cytokines as well as play an important role in chronic cardiovascular diseases [15]. However, HDL cholesterol could reduce the expression of CD11b and other adhesion molecules on monocytes and damaged endothelial cells, inhibiting the activation and proliferation of monocytes and thus exerting both anti-inflammatory and antioxidant effects [16]. Recently, the combined circulating monocyte count and serum HDL cholesterol in one fraction, termed ‘MHR’, was found to be associated with a poor cardiovascular prognosis and emerged as an independent predictor of major cardiovascular events in patients with chronic kidney disease [5]. As a quantitative index of inflammation and oxidative stress, MHR reflects the degree of inflammation and oxidative stress to a certain extent and was found to not only be a predictor of cardiovascular diseases in patients with chronic obstructive pulmonary disease [17] but also to have a certain value in predicting the occurrence of AF after coronary artery bypass grafting (CABG) [18]. The study included 311 patients who had undergone CABG, and 71 patients developed AF after surgery. The MHR in the group with AF was significantly higher than that of the control group. Additionally, the MHR had also been found as an independent risk factor for AF recurrence after cryoballoon-based catheter ablation. Canpolat et al. [6] enrolled 402 patients who had undergone ablation, and a high MHR before surgery could predict AF recurrence after a mean follow-up time of 20.6 ± 6.0 months. In our present study, the increased MHR along with the LAD before radiofrequency ablation is a strong and independent predictor of AF recurrence during the follow up. In clinical practice, we could evaluate the recurrence risk of AF using the preoperative LAD and MHR to select appropriate patients for the procedure, reduce unnecessary interventions and early identify high-risk patients with recurrence, and provide corresponding strengthening intervention measures, to improve the long-term prognosis of the patients. Additionally, the MHR showed the same value of predicting AF recurrence as the LAD, which has been widely accepted as a risk factor for AF recurrence after ablation [19]. Additionally, the MHR was positively correlated with the LAD.

### Limitations

This study is a single-center retrospective study, and the conclusion needs to be further explored by multiple centers. Additionally, the sample size of this study is relatively small, and the postoperative follow-up time is limited. Moreover, the recurrence of asymptomatic AF may be ignored in the follow-up process, contributing to a certain bias in the detection of the postoperative recurrence of AF.

## Conclusion

In our study, we observed that the elevated preablation MHR was associated with an increased risk of the postoperative recurrence of AF. Additionally, the MHR independently predicted the late recurrence of paroxysmal AF after radiofrequency ablation, with the same predictive value as the LAD.

## Data Availability

The datasets analyzed during the current study are available from the corresponding author on reasonable request.

## Abbreviations

MHR: Monocyte/HDL ratio
AF: Atrial fibrillation
LAD: Left atrial diameter
ROC: Receiver operating characteristic curve
CI: Confidence interval
AUC: Area under the curve
LVEF: Left ventricular ejection fraction
CABG: Coronary artery bypass grafting
